# Predicting outcome in clinically isolated syndrome using machine learning

**DOI:** 10.1016/j.nicl.2014.11.021

**Published:** 2014-12-04

**Authors:** V. Wottschel, D.C. Alexander, P.P. Kwok, D.T. Chard, M.L. Stromillo, N. De Stefano, A.J. Thompson, D.H. Miller, O. Ciccarelli

**Affiliations:** a NMR Research Unit, UCL Institute of Neurology, Queen Square MS Centre, Queen Square, London, UK; b Department of Computer Science, Centre for Medical Imaging Computing, UCL, London, UK; c National Institute for Health Research (NIHR), University College London Hospital (UCLH), Biomedical Research Centre (BRC), UK; d Department of Neurological and Behavioral Sciences, University of Siena, Siena, Italy

**Keywords:** Support vector machines, MRI, Multiple Sclerosis, Clinically isolated syndrome

## Abstract

We aim to determine if machine learning techniques, such as support vector machines (SVMs), can predict the occurrence of a second clinical attack, which leads to the diagnosis of clinically-definite Multiple Sclerosis (CDMS) in patients with a clinically isolated syndrome (CIS), on the basis of single patient's lesion features and clinical/demographic characteristics.

Seventy-four patients at onset of CIS were scanned and clinically reviewed after one and three years. CDMS was used as the gold standard against which SVM classification accuracy was tested. Radiological features related to lesional characteristics on conventional MRI were defined a priori and used in combination with clinical/demographic features in an SVM. Forward recursive feature elimination with 100 bootstraps and a leave-one-out cross-validation was used to find the most predictive feature combinations.

30 % and 44 % of patients developed CDMS within one and three years, respectively. The SVMs correctly predicted the presence (or the absence) of CDMS in 71.4 % of patients (sensitivity/specificity: 77 %/66 %) at 1 year, and in 68 % (60 %/76 %) at 3 years on average over all bootstraps. Combinations of features consistently gave a higher accuracy in predicting outcome than any single feature.

Machine-learning-based classifications can be used to provide an “individualised” prediction of conversion to MS from subjects' baseline scans and clinical characteristics, with potential to be incorporated into routine clinical practice.

## Highlights

• SVMs predict the presence (or absence) of a second clinical attack in Multiple Sclerosis at 1- and 3-year follow-ups.
• SVM-based classification reaches 71.4 % accuracy, 77 % sensitivity and 66 % specificity for 1-year follow-up.
• Combinations of features give a higher accuracy than single features.

## 1 Introduction

Machine learning is an emerging area of computer science and artificial intelligence that provides an increasing variety of algorithms capable of learning patterns from input data to solve classification and prediction problems (Bishop, 2006). Support vector machines (SVMs) are well-established classification algorithms (Vapnik, 1995) and a popular choice due to their simplicity and high performance in a range of applications. In the context of medical imaging, SVMs have shown promise for binary classifications (e.g. disease vs. healthy status), on the basis of imaging characteristics (Ashburner and Klöppel, 2011). In this context, SVMs first learn the characteristics of, say, MRI scans in each of two groups; then, they use that knowledge to assign new brain scans, which have not been used in the training procedure, to one of the two groups. SVMs have been applied in this way to imaging data from a variety of neurological and psychiatric diseases to assist in the diagnostic process, including pre-symptomatic Huntington's disease (Klöppel et al., 2009), Alzheimer's disease (Klöppel et al., 2008a), autism spectrum disorder (Anderson etal., 2011), and major depressive disorder (Mwangi et al., 2012). A few studies have applied SVMs to data from patients with MS, suggesting that SVMs may become a useful tool for automatic classification of MS patients vs. healthy controls (Weygandt et al., 2011) and MS patients with different characteristics (such as patients with early MS vs. those with late MS) (Bendfeldt et al., 2012). A key question that is of direct clinical relevance, and is addressed in this study, is whether SVMs can be applied to MRI scans and clinical characteristics of patients with early features of Multiple Sclerosis (MS) to predict their prognosis.

For most patients with MS, the onset of their condition is with an episode of neurological disturbance, known as a clinically isolated syndrome (CIS) (Miller et al., 2012). About 30 % of patients with CIS present with a second clinical attack within 1 year from onset, leading to the diagnosis of clinically-definite MS (CDMS) (Miller et al., 2012). However, about 20 % of CIS patients do not convert to MS after two decades, even if they have an abnormal brain scan at onset (Fisniku et al., 2008). Therefore, individual patients presenting with CIS face the uncertainty of if and when a second relapse will occur.

Research into the predictors of clinical outcome in CIS has demonstrated that the number, location and distribution of asymptomatic white matter lesions on a brain scan at first presentation are associated with the risk of having a second clinical attack (Brex et al., 2002; Giorgio et al., 2013; Swanton et al., 2007; Tintore et al., 2006). For example, patients with CIS whose baseline scans fulfil 3 or 4 Barkhof criteria (i.e., the occurrence of gadolinium enhancing lesion, juxtacortical lesion, infratentorial lesion and periventricular lesion) (Barkhof et al., 1997) have an adjusted hazard ratio of 17 (95 % confidence interval (CI) 6.7–43.5) for clinical conversion to MS during a 7-year follow-up (Tintore et al., 2006). When dissemination in space criteria are considered (i.e., at least one lesion in at least two typical locations: periventricular, juxtacortical, posterior fossa, and spinal cord) (Polman et al., 2011), the likelihood ratio for CDMS in patients with CIS is 2.1 (95 % CI 1.7–2.7) during a 3-year follow-up, with a sensitivity of 85.9 % and specificity of 59.4 % (Swanton et al., 2007). Additionally, demographic and clinical characteristics at the onset of a CIS, such as younger age, female gender and multifocal neurological involvement, are also associated with a higher risk of developing MS in short-term (Miller et al., 2012).

These MRI and clinical factors are commonly used in clinical practice to counsel individual patients about their risk of developing CDMS, but they are not combined to provide an overall estimate of risk of conversion. Ideally, a person-specific “individualised” risk of a second clinical relapse would be estimated, instead, based on an individual scan and clinical characteristics; this represents a crucial step in the improvement of patient management.

Therefore, the primary aim of this study was to determine whether SVMs can predict clinical conversion to MS (or the absence of clinical conversion) from CIS during one- and 3-year follow-ups. A secondary aim was to highlight lesional and clinical/demographic features that appear important to the prediction of CDMS.

## 2 Methods

### 2.1 Subjects

This is a retrospective study. None of the patients studied was on disease modifying treatments. Seventy-four patients were scanned after a mean of 6.15 weeks (SD 3.4) from the onset of a CIS, and clinically reviewed after 1 year; 70 patients attended a follow-up visit after 3 years. This represents a subgroup of a larger cohort recruited between 1995 and 2004; to be included in the present study, at least one demyelinating lesion must have been visible on baseline scans, and those scans, together with their corresponding lesion masks, had to be available for inclusion in this project. Additionally, clinical data at one and three year follow-ups must have been available.

In all patients, clinical and demographic information at onset, including type of CIS presentation (i.e., spinal cord, optic nerve, brainstem, multifocal), age, gender, and Expanded Disability Status Scale (EDSS) at baseline, was recorded. Clinical conversion to MS due to the occurrence of a second clinical attack attributable to demyelination of more than 24 hours in duration and at least 4 weeks from the initial attack was noted at each follow-up review. Informed consent from each patient and ethical approval by the local ethics committee was obtained prior to the study. The patients' characteristics are summarised in Table 1.

### 2.2 MRI acquisition and pre-processing

**Fig. 1:**
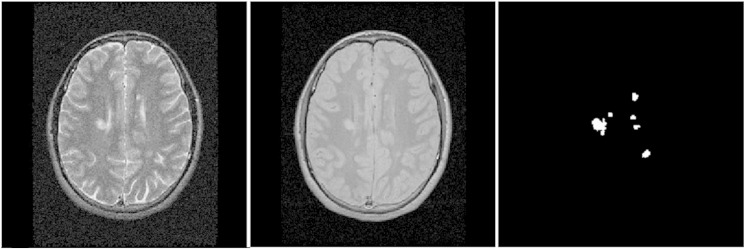
Example of T2 and PD weighted images and corresponding binary lesion mask. Axial T2 weighted image (left), and proton density (PD) weighted image (centre), showing hyperintense white matter lesions; the corresponding binary lesion mask (right) was used to obtain the lesion features entered into the SVM analysis.

Baseline MRI protocol was undertaken using a 1.5 T GE Signa MRI scanner. A brain FSE dual echo sequence, yielding proton density (PD) and T2 weighted images (TR = 3200 ms, TE = 15/90 ms, contiguous 3 mm axial slices, in-plane resolution 0.9375 × 0.9375 mm^2^) was obtained. Binary lesion masks were created by one experienced neurologist marking the lesions in the PD images of all patients, using the corresponding T2 images as reference (Fig. 1), with an in-house semi-automated software.

All the PD and T2 images were spatially normalised to the MNI152 standard space T1 image using a diffeomorphic registration with NiftyReg (Modat et al., 2010) (http://cmic.cs.ucl.ac.uk/home/software/). The resulting transformation parameters were applied to the lesion masks allowing us to define a spatial reference point that can be used to calculate distance-based features for all patients.

### 2.3 Classification analysis

**Fig. 2:**
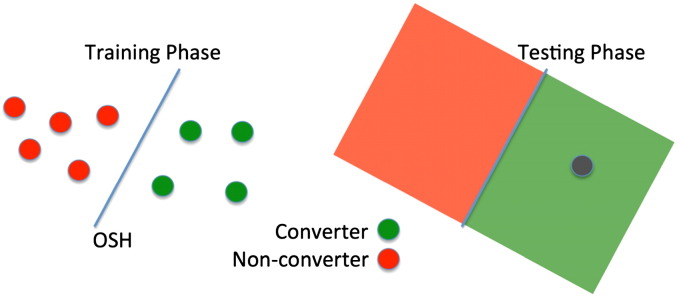
Illustration of one permutation within a leave-one-out cross-validation using support vector machines. Training phase: data points with known labels are used to create an optimal separating hyperplane (OSH). Testing phase: previously unseen data point (grey) is assigned a label (converter) based on the position relative to the OSH.

In this study, Support Vector Machines (Vapnik, 1995; Vapnik, 2008) were used for binary classification. SVMs are supervised learners that work in two phases. In the training phase, a subset of the available data points as well as their associated classes is used to iteratively find a linear boundary or hyperplane that separates the two classes optimally. In the testing phase, new, previously unseen data points in the same space as the training points are classified depending on their position relative to the boundary as shown in Fig. 2. In this study, each data point is a multidimensional vector consisting of a relatively small number of a priori defined features but, generally, data points can contain any information associated with the respective subject including much larger feature sets, such as all MRI voxel intensities, as in e.g. Klöppel et al. (2008a) or Bendfeldt et al. (2012).

#### 2.3.1 Feature definition

Each feature represents one dimension of the data points used for training and testing. We selected a priori demographic/clinical features and lesion features, which were chosen to capture information on white matter lesion load, distribution, size, and signal intensity. The mean and SDs of all features are shown in Supplementary Table 1.

The four demographic/clinical features are age, gender, type of CIS, and EDSS at baseline. The gender was coded with 1 referring to male and 0 to female. The CIS type was coded according to 1=optic neuritis, 2=spinal cord, 3=brainstem, and 4=other. This coding was arbitrarily chosen. A permutation of this numbering, however, has little effect and reduces the accuracies of the best feature combinations by a maximum of 1.7 % (detailed results not shown). The following 8 lesion features were extracted from the PD/T2 images and lesion masks of each patient:

(1) Lesion count: this feature reflects the total number of lesions in the brain, extracted from the native lesion masks; it was computed using the original binary lesion masks and an 18-neighbourhood for voxel connectivity.
(2) Lesion load: this feature reflects the total lesion volume, in voxels, extracted from the native lesion masks
(3) Average lesion PD intensity: this feature reflects the average PD intensity of the lesional voxels marked in the native lesion masks.
(4) Average lesion T2 intensity: this feature reflects the average T2 intensity of the lesional voxels included in the native lesion masks.
(5) Average distance of lesions from the centre of the brain: this feature gives the average distances between all lesional voxels and the centre of the brain (defined as the central voxel of the MNI152 registration template), providing information on how spread out the lesions were on the registered images [Supplementary Fig. 1].
(6) Presence of lesions in proximity of the centre of the brain: this binary feature is 1 if there are lesions within a cube of 1 cm^3^ centred around the central voxel of the SPM template, or 0 if no lesions were in the central box. This feature was selected because of the evidence that lesions located in the corpus callosum, which is a midline brain structure, are useful in predicting conversion to CDMS in addition to the Barkhof criteria (Jafari et al., 2009).
(7) Shortest horizontal distance of a lesion from the vertical axis of the brain: this feature measures the shortest distance of a lesion's centroid (centre of mass) from the intersection of the midsagittal and midcoronal planes of the image. This feature represents an additional way of reflecting the distance of the lesions from the centre of the image.
(8) Lesion size profile: this feature reflects the distribution of lesion sizes. All lesions in native space were sorted according to their size and divided into three groups of equal length representing small (1–15 voxels), medium (16–36 voxels) and large (37+ voxels) lesions which give reasonably similar numbers in each category over the whole data set (see Supplementary Table 1).

#### 2.3.2 Leave-one-out cross-validation

The conversion to MS on the basis of a second clinical episode was the gold standard against which the SVM's classification accuracy was tested. The SVM classification was performed using the functions *svmtrain* and *svmclassify* from the MATLAB (2012a) statistics toolbox. Different feature combinations of the twelve lesion/demographic/clinical features were tested using a recursive algorithm, subsequently adding the best performing feature from each individual feature alone, pairs etc. A polynomial kernel (K(**x,y**) = (x^T^y + c)*^d^*) of degrees *d* from 1 to 5 was used; this includes the widely used linear kernel, which is a polynomial kernel of degree one, but also allows the classifier to use more complex models. We limit the degree to 5 to avoid overfitting. Parameter optimisation was performed with an inherent sequential minimal optimisation (SMO) with 10 million iterations to allow for convergence.

The unbalanced group sizes of 22 converters vs. 52 non-converters and 31 converters vs. 39 non-converters for one and three years respectively can lead to a bias of the hyperplane weighting towards the larger group, and, in addition, often results in a high sensitivity and a low specificity or vice versa. Therefore 100 random samples were selected from the larger group with size equal to the smaller group. In the case of the 1-year follow-up this means that 22 non-converters were randomly selected from the whole set of 52 non-converters in order to match the group size of the 1-year converters. This procedure was repeated 100 times to allow for the estimation of a confidence interval from these bootstraps and give a better idea of how the results will generalise to the whole cohort. The resulting cohorts of 44 (22 converters and 22 non-converters) and 62 (31 converters and 31 non-converters) for 1 and 3 years respectively were then used to train and test an SVM using the common leave-one-outcross-validation (LOO-CV) (Young et al., 2013). In a LOO-CV for our 1-year follow-up 43 out of 44 patients are used in the training phase to calculate an optimal separating hyperplane (OSH). The remaining patient is then classified using this OSH as shown in Fig. 2. The training and testing samples are permuted until every patient was used for testing once. The nature of LOO-CV implies that in each individual training step the classes are slightly imbalanced (i.e., 21 vs. 22 or 30 vs. 31) as one patient is always left out of the training cohort. This procedure, however, is performed for both classes in the exact same way so that this effect can be neglected.

#### 2.3.3 Feature combinations

The performance of the SVMs was investigated by computing the accuracy of the classification for each individual feature as well as a feature combination obtained from a feature-selection procedure. Accuracy was defined as the percentage of patients correctly classified as either converters or non-converters; sensitivity was defined as the percentage of patients with CDMS correctly classified as converters, while specificity as the percentage of patients without CDMS correctly identified as non-converters; positive predictive value (PPV) was defined as the proportion of patients classified as converters who were truly converters, while negative predictive value (NPV) as the proportion of subjects classified as non-converters who were truly non-converters. We compute these values for each of the 100 bootstrap samples and report mean values, CIs and ranges over the 100 resulting values.

**Fig. 3:**
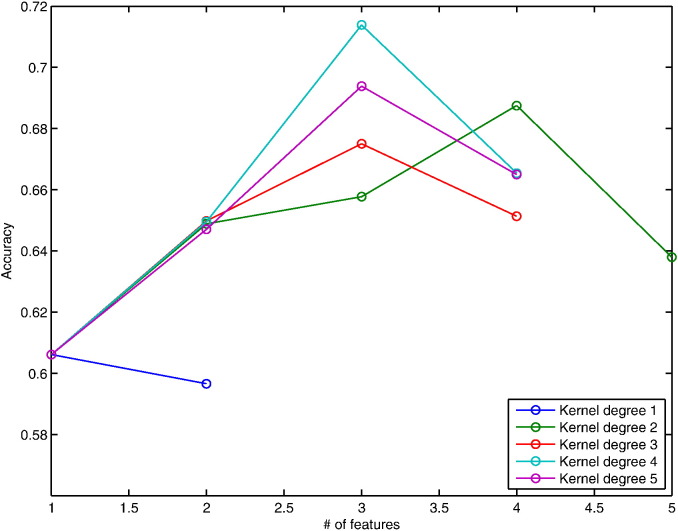
Accuracies of forward RFE for 1-year prediction. Plot showing the development of accuracies after recursively adding features in order to find the most predictive combination for conversion within 1 year.

**Fig. 4:**
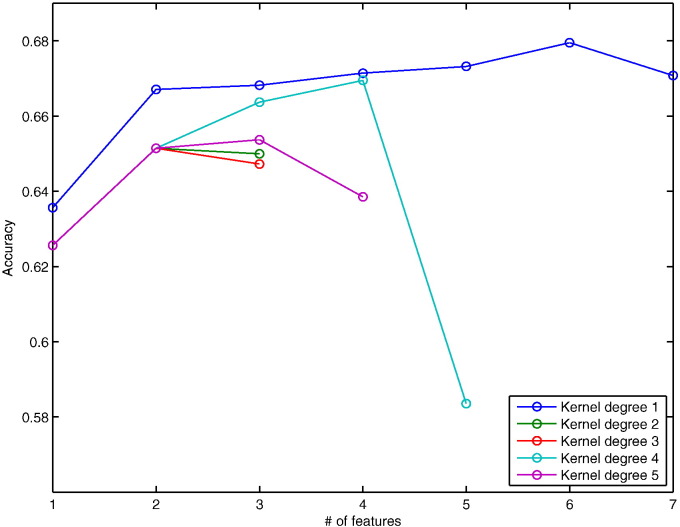
Accuracies of forward RFE for 3-year prediction. Plot showing the development of accuracies after recursively adding features in order to find the most predictive combination for conversion within 3 years.

A forward recursive feature elimination (fRFE) algorithm was used to combine features subsequently while testing their classification performance. We start with every individual feature by itself and identify the one with the highest classification accuracy averaged over the 100 bootstraps. Then, one of the remaining 11 features is added to identify the best combination of two features. This procedure is repeated subsequently to test larger feature combination sets until the obtained classification accuracy does not increase anymore (Figs. 3 and 4). The features in the combination associated with the highest classification accuracy are reported (Table 2).

## 3 Results

### 3.1 Classification results using feature combinations

The demographic and clinical characteristics of patients are summarised in Table 1. 30 % and 44 % of patients developed CDMS within one and three years respectively. Table 2 presents the average results from the 100 bootstraps at 1 and 3 years. The highest average accuracy at 1 year was 71.4 %, which means that, on average, SVMs correctly predicted CDMS (or the absence of clinical conversion) in 71.4 % of patients (Fig. 3), with a sensitivity of 77 % (i.e., 77 % of patients with CDMS at 1 year were identified as converters) and specificity of 66 % (i.e., 66 % of patients without CDMS at 1 year were identified as non-converters) obtained with a polynomial degree of 4. The accuracy range of the 100 bootstraps was 52–84 % with a 95 % confidence interval (CI) of 58–84 %. Similarly, the highest average prediction accuracy at 3 years was 68 % (Fig. 4), with a sensitivity of 60 % and specificity of 76 % obtained with a polynomial kernel of degree 1. The PPV and NPV were 70 % and 74 %, and 72 % and 65 % for and 1 and 3 years respectively (Table 2). The accuracy range for the 3-year follow-up was 61–74 % with a 95 % CI of 61–73 %.

No specific patterns or common characteristics were observed in patients who were not correctly classified as converters or non-converters on the basis of their baseline scans and clinical characteristics.

### 3.2 Lesional and clinical features most relevant to the classification

The features in the best combination for prediction of conversion to CDMS (or not) at 1 year were: type of presentation, gender and lesion load. At 3 years, the features in the best combination were: lesion count, PD intensity, mean distance from lesions to the centre of the brain, shortest distance from lesions to the vertical axis of the brain, EDSS, and age (Table 2).

**Fig. 5:**
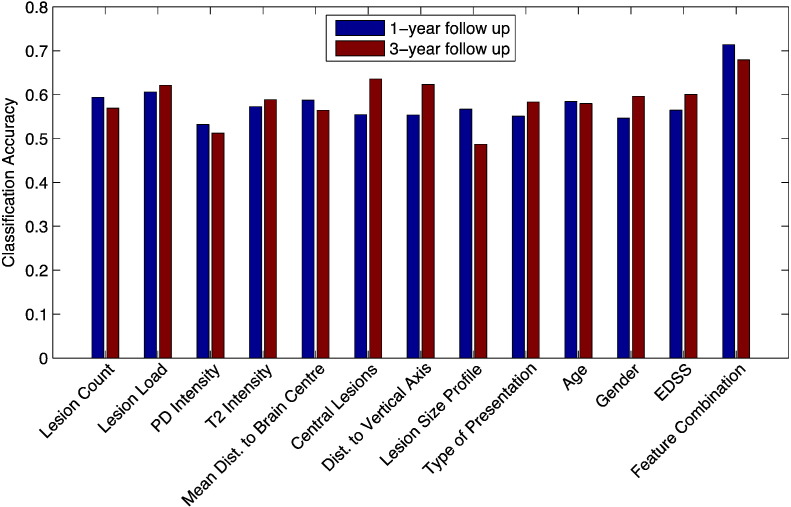
Performance of single features vs. feature combination. Bar plot showing the classification accuracy of all individual features vs. the best combination of features obtained with SVMs.

These combinations achieved an approximately 10.8 % (1 year) and 4.4 % (3 years) higher accuracy than that obtained with the best performing single feature (Fig. 5).

## 4 Discussion

SVMs correctly classified CDMS (or the absence of clinical conversion) at one and three years in 71.4 % and 68 % of CIS patients respectively using individually labelled brain scans and associated clinical information on average over 100 bootstraps with balanced training data sets using leave-one-out cross-validation for testing. At present, patients who present with CIS are told that they have a long-term risk for CDMS of 60–80 % when white matter lesions are seen on the brain scans, and on the basis of the number and location of brain lesions have a low, medium and high conversion risk to MS (Tintore et al., 2006). Female patients are told they have a relative risk of developing CDMS of 1.20 (95 % CI 0.98−1.46) compared with males (Dobson et al., 2012). However, there are limitations in accuracy (sensitivity and specificity) when extrapolating radiological and clinical predictors from these group studies to individual cases in routine clinical practice. The main potential of the SVM-based classification is that it can be used for a single subject (or individualised) prediction of clinical conversion to MS. This may lead to a more tailored prognosis, which, in turn, would translate into more timely and better-informed treatment choices. In addition, accurate prediction of prognosis from individual subjects' scans may also have a beneficial impact on research, by helping to select patients for clinical trials and research studies.

### 4.1 SMV-based classification

The 71.4 % and 68 % average classification accuracy obtained with SVMs is slightly lower than those reported in previous applications of SVMs to other neurological diseases (Klöppel et al., 2008b; Klöppel et al., 2009; Mwangi et al., 2012). However, it is important to note that the classification of patients into those who will develop MS within a short-term follow-up and those who will not is a more challenging problem than classifying patients vs. healthy subjects (Hackmack et al., 2012; Weygandt et al., 2011), since some of the patients in the non-converter group may still develop MS in the long-term. Studies on a similar classification task on patients with mild cognitive impairment (MCI) obtained lower or similar accuracies in the range from 62 % to 75 % for distinguishing between MCI-stable patients and MCI patients who convert to Alzheimer's disease (Young et al., 2013).

### 4.2 Lesional and clinical features most relevant to the classification

By considering together the results of the features associated with the highest accuracy of prediction, we found that lesion load and count were selected by the fRFE-SVMs to obtain a high classification accuracy rather than other features, such as lesion size. This is in agreement with previous papers (Miller et al., 2012). Interestingly, we found that the distance of lesions to the vertical axis of the brain was associated with a conversion to MS within 3 years, suggesting that lesion location may be an important predictor of future clinical attacks in CIS as suggested for the corpus callosum (Jafari et al., 2009), for the brainstem (Tintore et al., 2010), and for the corona radiata, optic radiation, and splenium of the corpus callosum (periventricularly) (Dalton et al., 2012). Specifically, a shorter distance of the lesions to the vertical axis of the brain was seen more often in converters than non-converters. The role of lesion location on clinical conversion to MS has been recently demonstrated by the association between a high lesion frequency (obtained by using the MRI lesion probability maps) in specific white matter regions and conversion to MS (Giorgio et al., 2013).

In addition to the imaging features, we included clinical and demographic features known to be relevant to the conversion to MS from CIS, such as age (Ruet et al., 2011) and gender (Dobson et al., 2012), and have confirmed that these are present in the combinations of features associated with the highest accuracy for classification at three and one year respectively; younger, female patients convert to MS more often than older, male patients. For a short-term conversion to MS, type of CIS seems to be relevant, as more patients with the spinal cord type convert within 1 year. Overall, the performance obtained with the use of single features individually to predict outcome was lower than that using combinations of both MRI and clinical/demographic features, suggesting that clinical and demographic characteristics may become crucial discriminative markers that need to be combined with imaging features to obtain the best possible accuracy for classification of individual patients.

Although more complex models (high degree polynomial kernels) with more input dimensions (combinations with more features) should always classify *training* data better than simpler models, our cross validation test considers, at least partially, generalisation to unseen test data so the identification of the best feature combinations is robust to overfitting. The fact that, the best performing feature combinations contain only a small number of features (3 and 6) and do not use the highest polynomial degree, even though we allow our model to use up to twelve features and polynomial kernels up to a degree of five, indicates that the higher classification accuracies obtained from the feature combinations compared to the individual features are not simply the result of using a more complex model.

### 4.3 Limitations and future studies

For classification tasks e.g. with SVMs it is important to make sure that a patient that has been used during the training phase is not used for testing as well. Ideally, this is achieved by having completely independent training and test sets to avoid any bias. However, this is not always possible, especially when the available data set is small as in the case of this study. The presented leave-one-out cross-validation provides a partial solution to this problem, but this generally introduces a positive bias in the accuracy. Since all feature combinations in this study were tested with the exact same methods, the comparison and ranking of the feature combinations remains valid, but the bias does affect the absolute values of the accuracy that each combination achieves; it is likely to be lower on unseen data.

An issue often debated relates to the choice of features that need to be selected to perform the experiments with machine learning techniques (Chu et al., 2012). A limitation of this work is that we only used features that we selected a priori and were associated with white matter lesions (visible on T2-weighted scans) that are known to be of value in the development of MS (Miller et al., 2012) and that discriminate between MS and healthy subjects (Hackmack et al., 2012). At present, these features are based on lesion masks, which are manually created by an observer, rather than the outputs of automated image analysis methods. It is assumed that the type of presentation is not directly correlated with the risk of conversion (Giorgio et al., 2013; Polman et al., 2008) but supports the SVM classification at 1-year follow-up. Future work will try to match or even surpass the SVM classification performance using purely automatically derived features, and features containing information on the different aspects of the imaging data (such as scale and directionality information (Hackmack et al., 2012)).

Additionally, it will be interesting to investigate whether classification accuracy improves if MRI features that reflect damage outside the MS lesions, such as those obtained with magnetisation transfer imaging (Audoin et al., 2006), are included. This is especially true since studies on MTR as an independent predictor for a second relapse are inconsistent (Gallo et al., 2007; Traboulsee et al., 2002). Other MS-related para-clinical abnormalities, including intrathecal synthesis of oligoclonal bands (Tintore et al., 2008), grey matter atrophy (Calabrese et al., 2011), and genetic factor (Kelly et al., 1993), which were not available in this cohort, may be predictors of conversion to MS, and future work will test whether they can improve SVM-based classification accuracy of converters vs. non-converters. This also applies to more clinically applicable features such as spinal cord lesions, which might be particularly important for patients with a non-spinal-cord type of presentation (Hutchinson et al., 2014; Sombekke et al., 2013), cortical lesions, which need additional DIR or PSIR MRI acquisition (Filippi et al., 2010) and Gd-enhancing lesions, which allow the diagnosis of MS in CIS patients without a follow-up MRI scan or a second attack (Polman et al., 2011; Rocca et al., 2008). In theory, the features used by SVMs can be potentially infinite, although computational time has to be limited to a reasonable period of time, and not all the features may be important to reach a high accuracy in the classification.

On the other hand, the fact that we used features provided by conventional (standard) brain imaging protocols and the most straightforward clinical/demographic features, which are available in any clinical centre, is an important advantage of our study, because it suggests that machine learning techniques can be used in centres that lack specialist research expertise and support the local physicians in their patient management.

The recursive feature elimination algorithm is a very common method to identify relevant features. However, the greedy nature of the search means that it often does not find the most predictive combination of features but only a local maximum. Another option is to search all possible combinations of features exhaustively. Although computationally expensive, the exhaustive search is feasible for our 12 features. Interestingly, this approach identifies the same feature combination as the fRFE for the 1-year follow up; for the 3-year follow up, it differs and finds: lesion count, lesion load, shortest horizontal distance of lesions from brain centre, age, gender and EDSS. The accuracy obtained with this combination was 5.5 % higher (73.5 %) than the fRFE result. However, while interesting to compare, the exhaustive search leads to a multiple comparisons problem since testing 2^12^ = 4096 different models on the same classification task is likely to identify a combination that spuriously performs well on this specific data set (of only 74 data points) so generalises less well to unseen data, so we believe that the fRFE performance is more indicative of what we can expect on unseen data. Our algorithm only adds one feature at each iteration that introduces the highest information gain; this means that the fRFE has an inherent control for redundant features. If two features contain the same information only one of them will be selected. This resulting feature set is not necessarily the only one informative about the classification task, since some highly correlated features may have been rejected. The result from the exhaustive search across the whole feature space in fact indicates that there is only one combination of our 12 features that leads to the reported accuracy values, although this may of course be a spurious effect from the limited size of the data set. Further study of the correlations among all features using a larger data set would be required to make strong statements about which feature combination is truly most informative.

The next step of this work is to confirm these findings in an independent (and larger) data set, which divides the data into training and testing sets; we are also interested in assessing whether SVMs can work across centres, so that the possibility of “exchanging” trained SVMs between clinical centres may be feasible for MS, as it is for other diseases (Klöppel et al., 2008a; Stonnington et al., 2010). Additionally, we will test whether better classification rates for progression of disability (or clinical outcome) may be obtained by including the temporal ordering of events (i.e., serial clinical and MRI scans), using novel algorithms, such as those we recently applied to Alzheimer's and Huntington's disease cohorts (Fonteijn et al., 2012).

## 5 Conclusion

We have shown that state-of-the-art machine learning techniques offer discrimination between CIS converters and non-converters on one to three year timescales and used the analysis to suggest lesional and clinical features whose combinations predict clinical conversion to MS. This computer-based technique has the potential to be used to inform clinical practice and research in MS and other neurological diseases.

The following are the supplementary data related to this article.

Supplementary Fig. 1 The centre of the brain is marked by the intersection of the white lines. These white lines are overlaid onto axial (left), sagittal (centre), and coronal images (right); the centre of the brain was used to calculate the average distance of lesions from the centre of the brain.

Supplementary Table 1 Lesional features extracted from baseline scans in respect to 1- and 3-year follow-up.

Supplementary data to this article can be found online at http://dx.doi.org/10.1016/j.inoche.2014.11.003.

## Figures and Tables

**Table 1:** Demographic and clinical characteristics of patients with CIS and at least one lesion at baseline.

	CIS at 1-year follow-up (total no. = 74)	CIS at 3-year follow-up (total no. = 70)
Gender (F/M)	49/25	47/23
Age, median, mean, median (range) years.	33.1, 34 (19–49)	33.2, 34 (19–49)
EDSS, median (range)	1 (0–8)	1 (0–8)
Type of onset, no (number of converters).	Brainstem/cerebellum = 6 (1)	Brainstem/cerebellum = 5 (1)
Spinal cord = 4 (4)	Spinal cord = 4 (4)
Optic neuritis = 64 (17)	Optic neuritis = 61 (26)
Others = 0 (0)	Others = 0 (0)
No. of patients with different number of lesions	Up to 3 lesions = 14	Up to 3 lesions = 13
More than 3 and up to 10 lesions = 23	More than 3 and up to 10 lesions = 23
More than 10 lesions = 37	More than 10 lesions = 34
Converters at follow-up, no. (%)	22 (30 %)	31 (44 %)

**Table 2:** The most predictive combination of features associated with the highest accuracy of prediction of conversion to CDMS at one and three years estimated from a forward RFE. Accuracy, sensitivity, specificity, PPV and NPV are average values of 100 bootstraps.

	1 year	3 years
**MRI features**		
Lesion count		●
Lesion load	●	
Average lesion PD intensity		●
Average lesion T2 intensity		
Average distance of lesions from the centre of the brain		●
Presence of lesions in proximity of the centre of the brain		
Shortest horizontal distance of a lesion from the vertical axis		●
Lesion size profile		
**Clinical features**		
Type of presentation	●	
Age		●
Gender	●	
EDSS at onset		●
**SVM-based classification**		
Polynomial degree	4	1
Accuracy (%)	71.4	68.0
Range (%)	52–84	61–74
95 % CI	58–82	61–73
Sensitivity (%)	77	60
Specificity (%)	66	76
PPV (%)	70	72
NPV (%)	74	65

CI = confidence interval; PPV = positive predictive value; NPV = negative predictive value.
