# Supplementary figures and images for: Predicting outcome in clinically isolated syndrome using machine learning
Source: Neuroimage Clin. 2014 Dec 4;7:281–7. doi: 10.1016/j.nicl.2014.11.021 (PMC4297887; doi:10.1016/j.nicl.2014.11.021)

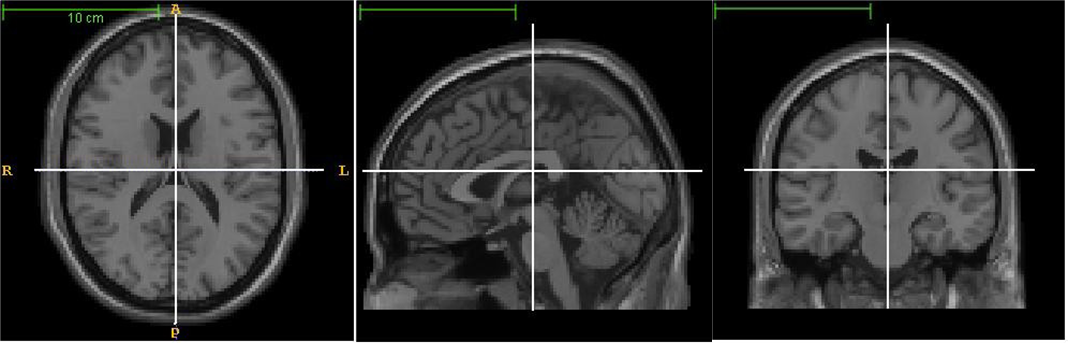

Supplement: Supplementary Fig. 1 — The centre of the brain is marked by the intersection of the white lines. These white lines are overlaid onto axial (left), sagittal (centre), and coronal images (right); the centre of the brain was used to calculate the average distance of lesions from the centre of the brain. [file mmc1.zip › YNC00400-mmc1.tif]
