# Supplementary material for: Predicting outcome in clinically isolated syndrome using machine learning
Source: Neuroimage Clin. 2014 Dec 4;7:281–7. doi: 10.1016/j.nicl.2014.11.021 (PMC4297887; doi:10.1016/j.nicl.2014.11.021)
Supplement: Supplementary Table 1 — Lesional features extracted from baseline scans in respect to 1- and 3-year follow-up. [file mmc2.docx]

**Supplementary Table 1. Lesional features extracted from baseline scans in respect to 1- and 3-year follow-up.**

| **1-year follow-up** |  |  |
| --- | --- | --- |
| **Feature** | **Mean** | **SD** |
| Lesion count | 17.20 | 18.85 |
| Lesion load (mm^3^) | 2257.5 | 3641.1 |
| Average lesion PD Intensity | 725.66 | 93.65 |
| Average lesion T2 Intensity | 408.93 | 63.24 |
| Average distance of lesions from the centre of the brain (mm^2^) | 19.2 | 3.4 |
| Presence of lesions in proximity of the centre of the brain | Yes in 37 patients, No in 37 patients |  |
| Shortest horizontal distance of a lesion from the vertical axis of the brain (mm^2^) | 7.9 | 5.9 |
| Lesion size profile:  No. of small lesions (below 16 voxels) | 5.72 | 7.12 |
| No. of medium lesions (between 16 and 36 voxel) | 5.73 | 6.32 |
| No. of large lesions (between 37 and 3306 voxels) | 5.76 | 6.81 |
| **3-year follow-up** |  |  |
| Lesion count | 16.53 | 18.06 |
| Lesion load (mm^3^) | 2209.5 | 3681.9 |
| Average lesion PD Intensity | 733.32 | 87.33 |
| Average lesion T2 Intensity | 414.00 | 60.11 |
| Average distance of lesions from the centre of the brain (mm^2^) | 19.2 | 3.4 |
| Presence of lesions in proximity of the centre of the brain | Yes in 35 patients, no in 35 patients |  |
| Shortest horizontal distance of a lesion from the vertical axis of the brain (mm^2^) | 7.8 | 5.4 |
| Lesion size profile  No. of small lesions | 5.57 | 6.97 |
| No. of medium lesions | 5.44 | 5.96 |
| No. of large lesions | 5.51 | 6.60 |
